# Comparative transcriptome analysis of two *Cercospora sojina* strains reveals differences in virulence under nitrogen starvation stress

**DOI:** 10.1186/s12866-020-01853-0

**Published:** 2020-06-16

**Authors:** Xin Gu, Shuai Yang, Xiaohe Yang, Liangliang Yao, Xuedong Gao, Maoming Zhang, Wei Liu, Haihong Zhao, Qingsheng Wang, Zengjie Li, Zhimin Li, Junjie Ding

**Affiliations:** 1 Jiamusi Branch of Heilongjiang Academy of Agricultural Sciences, Jiamusi, China; 2 Potato Research Institute, Heilongjiang Academy of Agricultural Sciences, Harbin, 150086 China

**Keywords:** *Cercospora sojina*, RNA-Seq, Comparative transcriptomics, Nitrogen starvation, Weighted gene co-expression network

## Abstract

**Background:**

*Cercospora sojina* is a fungal pathogen that causes frogeye leaf spot in soybean-producing regions, leading to severe yield losses worldwide. It exhibits variations in virulence due to race differentiation between strains. However, the candidate virulence-related genes are unknown because the infection process is slow, making it difficult to collect transcriptome samples.

**Results:**

In this study, virulence-related differentially expressed genes (DEGs) were obtained from the highly virulent Race 15 strain and mildly virulent Race1 strain under nitrogen starvation stress, which mimics the physiology of the pathogen during infection. Weighted gene co-expression network analysis (WGCNA) was then used to find co-expressed gene modules and assess the relationship between gene networks and phenotypes. Upon comparison of the transcriptomic differences in virulence between the strains, a total of 378 and 124 DEGs were upregulated, while 294 and 220 were downregulated in Race 1 and Race 15, respectively. Annotation of these DEGs revealed that many were associated with virulence differences, including scytalone dehydratase, 1,3,8-trihydroxynaphthalene reductase, and β-1,3-glucanase. In addition, two modules highly correlated with the highly virulent strain Race 15 and 36 virulence-related DEGs were found to contain mostly β-1,4-glucanase, β-1,4-xylanas, and cellobiose dehydrogenase.

**Conclusions:**

These important nitrogen starvation-responsive DEGs are frequently involved in the synthesis of melanin, polyphosphate storage in the vacuole, lignocellulose degradation, and cellulose degradation during fungal development and differentiation. Transcriptome analysis indicated unique gene expression patterns, providing further insight into pathogenesis.

## Background

Global soybean production is severely affected by *Cercospora sojina* Hara [1], which can infect all aerial organs of soybean plants [2]. It can result in more than 35% production loss under suitable climatic conditions [3], it also has strong infectivity and physiological race differentiation [4]. The emerging races have considerable virulence, which is the main reason for the loss of resistance of resistant soybean cultivars [5]. The use of resistant cultivars is the most effective strategy for the disease control, and the identification of *C. sojina* races provides a foundation for disease resistance breeding. Pure isolates were inoculated onto the leaves of differential soybean cultivars for identifying physiological races by phenotypes. At present, each major soybean-producing country uses its own local soybean cultivars to identify its own races, which is not conducive to disease communication and cooperation [1]. The climatic conditions in Northeast China are suitable for *C. sojina* dispersion, with the worst damage being recorded in Heilongjiang and Jilin provinces [6]. In recent years, a new strain, namely, Race 15 has emerged in Heilongjiang province. It not only exhibits an increasing frequency of isolation from the field but also shows high virulence. Its emergence has led to the loss of resistance in some soybean varieties resistant to Race 1 [7, 8]. Therefore, it is of great significance to clarify the molecular mechanisms of the infection process between virulent *C. sojina* strains.

Mycotoxins are toxic products excreted by *Cercospora* spp. and are widely considered to be key determinants that promote pathogenicity or virulence, as the virulence and pathogenicity of cercosporin-deficient mutants are considerably weakened [9]. However, it is uncertain whether *C. sojina* secrets cercosporin [10]. A previous study [11] analyzed the transcriptome of the *C. sojina* strain Race 1 under nitrogen starvation stress. During the early stage of infection, the genes involved in pigment biosynthesis were significantly upregulated, and eight cercosporin biosynthesis genes were screened. Amino acid sequence alignment showed that they shared the same tandem order with *Cercospora nicotianae*. Pigments are important secondary metabolites that allow pathogens to successfully infect hosts [12]. The results of this previous study implied that pigments play a pivotal role in the virulence of *C. sojina*. In previous pilot studies, only one CBM protein, CBM1, was found in the *C. sojina* Race 1 genome [11], which is significantly lower than other plant pathogenic fungi [13]. CBM is mainly derived from fungi and is primarily involved in cell wall hydrolysis. CBMs anchor the catalytic region of the enzyme to insoluble cellulose, enabling it to attach to plant cell walls and possibly improve the efficiency of the enzymatic digestion of plant cell walls [13]. The deficiency of CBMs in *C. sojina* may also slow down the infection progress, which may hinder the collection of sufficient samples for transcriptome sequencing. However, nitrogen starvation treatments can mimic the physiology of the pathogen during infection [14]. Nitrogen starvation is one of the main stresses inflicted on the host following pathogen infection [15] and greatly affects protein turnover, growth, and the cell cycle in many fungi, yeast, and bacteria [16, 17]. Nitrogen stress is one of the reasons for the large number of genes induced during *Magnaporthe grisea* infection [18]. The secreted products which were isolated from culture filtrates of *Magnaporthe grisea* under nitrogen starvation caused rice leaf senescence within 48 h and were also conducive to the formation of appressorium and enhanced pathogenicity levels [17, 18]. A transcriptome study under nitrogen starvation stress on Race 1 showed that secondary metabolism-related genes, including PKS, NRPS, and NRPS-like genes, which are related to mycotoxin synthesis, were significantly upregulated. Concurrently, a large number of cell wall-degrading enzymes were also found to be significantly upregulated; all of which may be related to pathogenicity [11]. While a previous study focused on the analysis of gene expression during *C. sojina* infection [11], our study focused on the DEGs associated with pathogenicity during infection between two strains with different virulence.

In this study we compared the transcriptomes of the highly virulent Race 15 strain and mildly virulent Race1 strain grown in nitrogen-deficient medium and nitrogen-containing medium using RNA sequencing (RNA-Seq) analysis. The mycelia were sequenced, and important virulence-related DEGs were identified. Functional categorization of the DEGs and virulence-related genes was conducted to reveal the various metabolic pathways involved in the response to infection. Weighted gene co-expression network analysis (WGCNA) was then used to identify co-expressed gene modules and assess the relationship between gene networks and phenotypes. Although many studies on fungal transcriptomes exist, no comparative transcriptomic studies have been conducted on strains of *C. sojina* differing in virulence. The results of this study help elucidate the molecular mechanisms of *C. sojina* infection and the causes of virulence differences in mildly and highly virulent strains.

## Results

### Virulence evaluation of the races and sequencing statistics

**Fig. 1:**
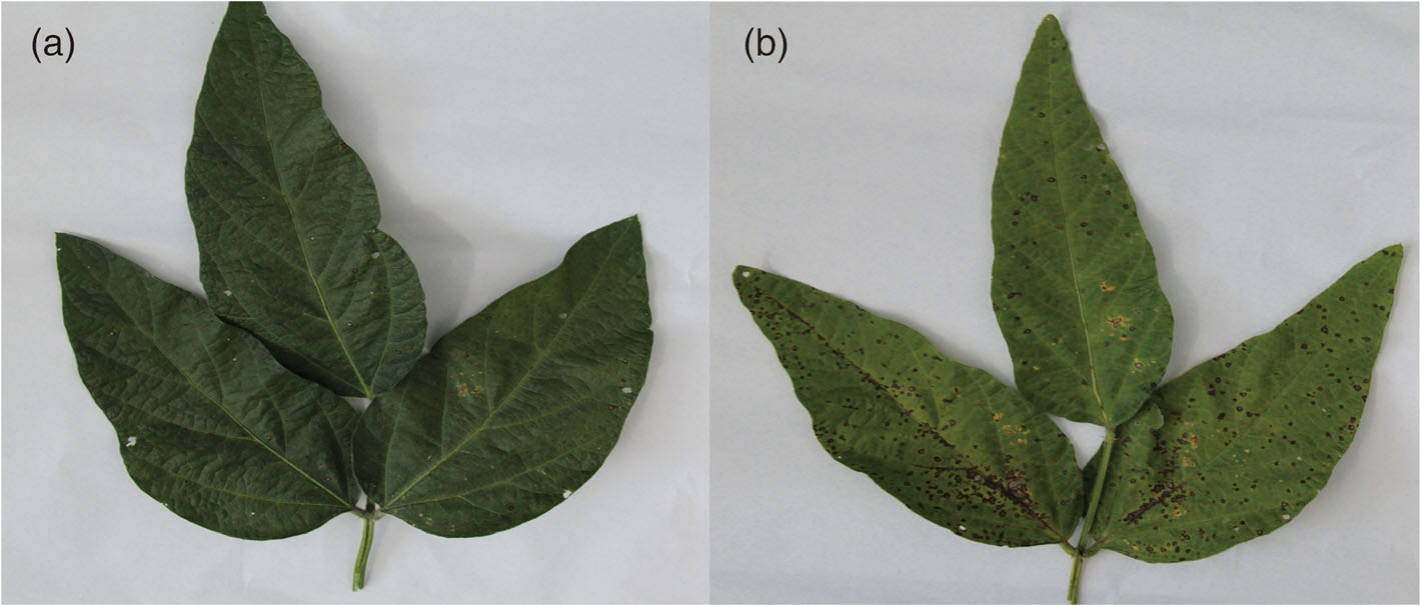
Comparison of the pathogenicity of different virulent strains of *C. sojina*, **a** Race15, and **b** Race1

The results showed that the disease index of Race 15 was 71.55 ± 0.59, which was much higher than that of Race 1 at 23.11 ± 2.12 (Fig. 1). The mycelia were grown in nitrogen-deficient medium and nitrogenous medium for 24 h, following which total RNA was extracted, and gene expression analysis by quantitative real-time (qRT)-PCR was carried out after transfection. A total of 487.58 million clean reads were obtained after filtering. The amount of clean bases of each sample exceeded 6,015,138,900 bp. More than 97.18% of the reads possessed quality values of Q ≥ 20, and the GC content was more than 54.85%, indicating that the data were suitable for downstream analyses. A total of 121.62 million, 121.94 million, 120.54 million, and 123.48 million clean reads from the Race 1 and Race 15 transcriptome libraries grown in nitrogenous medium (marked as Race1-CK and Race15-CK) and in nitrogen-deficient medium (marked as Race1-LN and Race15-LN), respectively (Table 1). The alignment results showed that 84.02–87.49% of clean reads from all of the 12 samples could be mapped to the reference genome. On average, approximately 17.02 million (83.99%) Race1-CK reads, 17.10 million (84.16%) Race1-LN reads, 17.09 million (85.06%) Race15-CK reads, and 17.15 million Race15-LN (83.34%) reads were uniquely mapped to the reference genome with HISAT2.

**Table 1:** Statistics of the transcriptome sequencing results

Sample Name	Clean Reads	Clean bases	Q20 (%)	GC (%)	Uniquely mapped	Total mapped
Race1_CK_24hA	40,961,854	6,144,278,100	98.08%	55.33%	17,103,260 (85.30%)	20,050,463(87.34%)
Race1_CK_24hB	40,402,594	6,060,389,100	97.18%	55.34%	17,187,857 (85.25%)	20,160,846(87.49%)
Race1_CK_24hC	40,256,508	6,038,476,200	98.02%	55.02%	16,972,254 (84.62%)	20,058,021(86.60%)
Race1_LN_24hA	41,539,426	6,230,913,900	98.04%	54.94%	17,406,685 (83.91%)	20,744,685(86.33%)
Race1_LN_24hB	40,143,194	6,021,479,100	98.02%	54.85%	16,631,562 (81.91%)	20,303,736(84.02%)
Race1_LN_24hC	40,254,896	6,038,234,400	98.11%	55.19%	17,422,073 (84.19%)	20,692,555(86.70%)
Race15_CK_24hA	40,100,926	6,015,138,900	98.23%	55.05%	17,136,290 (83.67%)	20,480,927(85.57%)
Race15_CK_24hB	40,321,692	6,048,253,800	98.30%	55.00%	16,769,650 (83.01%)	20,201,297(84.99%)
Race15_CK_24hC	40,116,042	6,017,406,300	98.27%	55.15%	17,168,804 (85.30%)	20,128,254(87.29%)
Race15_LN_24hA	41,489,370	6,223,405,500	98.04%	55.18%	17,484,097 (84.18%)	20,769,713(86.35%)
Race15_LN_24hB	40,607,472	6,091,120,800	98.06%	55.19%	16,877,992 (84.09%)	20,071,597(86.47%)
Race15_LN_24hC	41,385,110	6,207,766,500	97.99%	55.00%	16,948,292 (84.20%)	20,127,448(86.39%)

### Identification of DEGs

Four comparison groups were constructed for the samples of Race15 and Race1 grown in different media: Race1-LN vs. Race1-CK, Race15-LN vs. Race15-CK, Race15-LN vs. Race1-LN, and Race15-CK vs. Race1-CK. Upon comparison of the strain Race1 grown in different media, significantly differentially expressed transcripts were noted, with 378 DEGs upregulated and 294 DEGs downregulated among the 672 DEGs in the Race1-LN vs. Race1-CK group (Additional file 1: Table S1). When comparing the strain Race15 grown in different media, 124 DEGs were upregulated and 220 DEGs were downregulated among the 344 DEGs in the Race15-LN vs. Race15-CK group (Additional file 2: Table S2). It was obvious that there were approximately two-fold more DEGs associated with Race1 than Race15 after nitrogen starvation stress. The results indicated that nitrogen starvation stress had a greater effect on Race1 than on Race15.

**Fig. 2:**
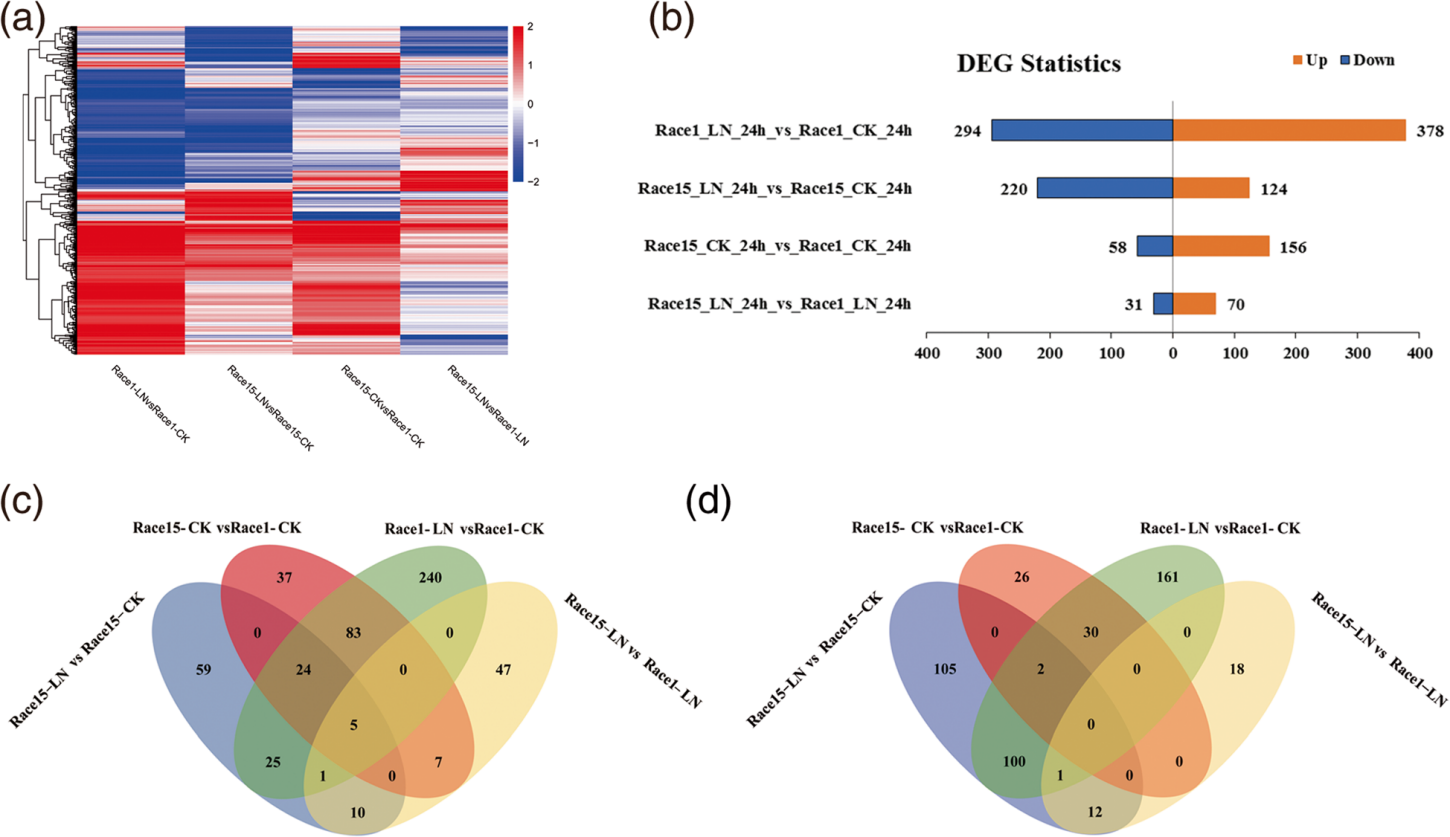
Summary of the differentially expressed genes among the four comparison groups. **a** Hierarchical heatmap showing the transformed expressional values for the transcripts. Red indicates upregulation and blue downregulation; **b** Differentially expressed genes statistics in the four comparison groups; **c** Venn diagrams for upregulated differentially expressed genes in the four comparison groups; and **d** Venn diagrams for downregulated differentially expressed genes in the four comparison groups

There were 70 and 31 DEGs that were upregulated or downregulated among the 101 DEGs in the Race15-LN vs. Race1-LN group (Additional file 3: Table S3), and 156 and 58 DEGs that were upregulated or downregulated among the 214 DEGs in the Race15-CK vs. Race1-CK group (Additional file 4: Table S4). It was also clear that there were more DEGs in Race15-CK vs. Race1-CK than in Race15-LN vs. Race1-LN. This result indicated that there were transcriptional differences between the highly and mildly virulent strains before nitrogen starvation stress. The DEGs between the sample groups were then visualized using hierarchical clustering and Venn diagrams (Fig. 2).

### GO and KEGG analysis of the DEGs

**Fig. 3:**
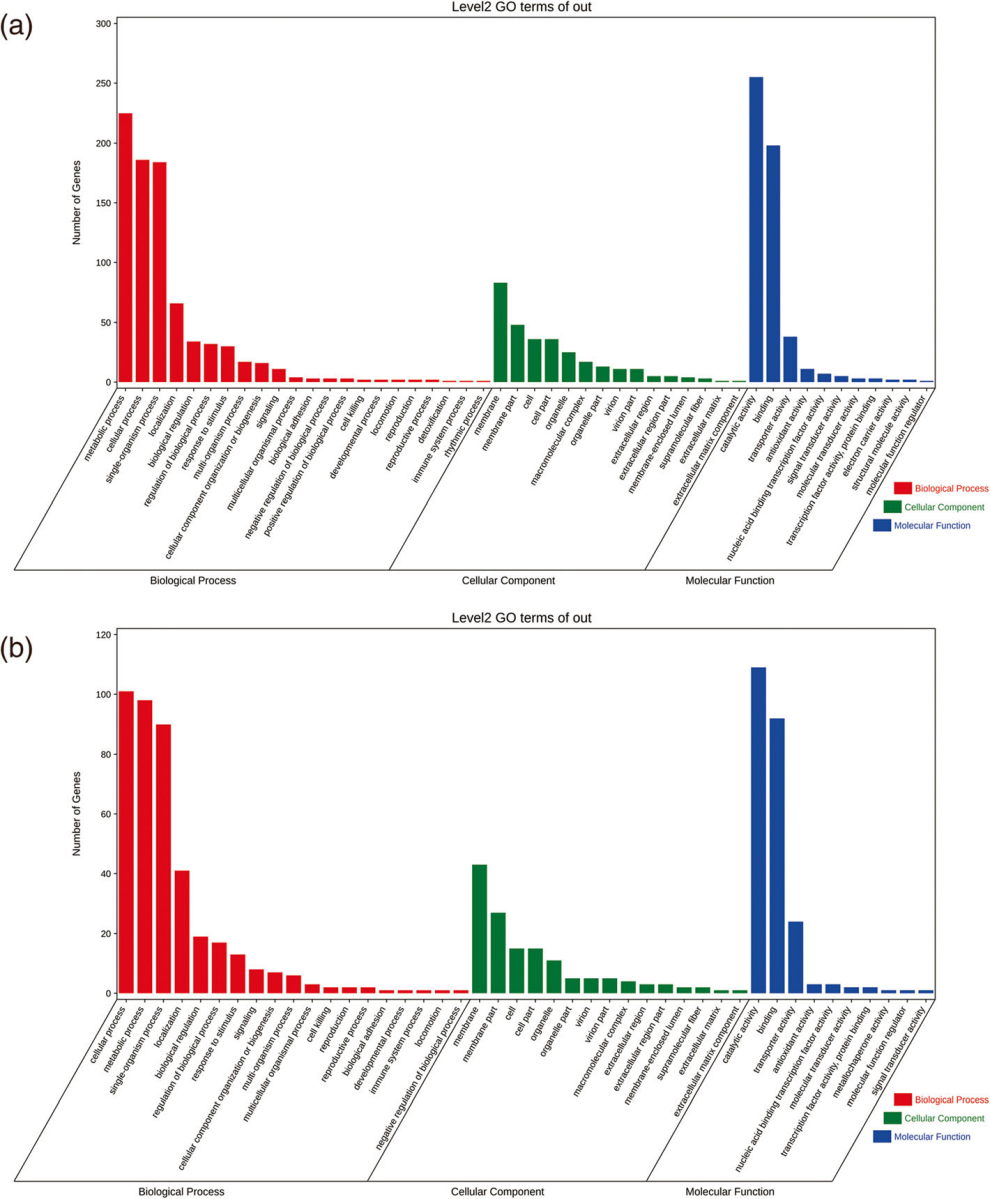
The most significantly-enriched GO terms of the differentially expressed genes from the four comparison groups: **a** Race1-LN vs. Race1-CK and **b** Race15-LN vs. Race15-CK down

Gene Ontology (GO) enrichment analysis indicates the biological functions that are significantly associated with differentially expressed transcripts. The 763 DEGs in the Race1-LN vs. Race1-CK group separated into three main categories, including 117 DEGs belonging to 17 GO groups based on cellular components, 350 DEGs belonging to 20 groups based on molecular functions, and 296 DEGs belonging to 33 groups based on biological processes. We further identified over-represented GO term categories (*p* < 0.05) in the DEGs of the Race1-LN vs. Race1-CK group and classified these terms into 48 categories. For biological processes, the dominant categories were single-organism process (GO: 0044699) with 88 DEGs, single-organism metabolic process (GO: 0044710) with 68 DEGs, establishment of localization (GO: 0051234) with 41 DEGs, transport (GO: 0006810) with 41 DEGs, and localization (GO: 0051179) with 41 DEGs. For molecular functions, the dominant categories were catalytic activity (GO: 0003824) with 157 DEGs, oxidoreductase activity (GO: 0016491) with 54 DEGs, hydrolase activity (GO: 0016787) with 53 DEGs, oxidoreductase activity (GO: 0016491) with 37 DEGs, and transporter activity (GO: 0005215) with 29 DEGs. For cellular components, the dominant categories were membrane (GO: 0016020) with 52 DEGs, membrane part (GO: 0044425) with 30 DEGs, integral component of membrane (GO: 0016021) with 28 DEGs, intrinsic component of membrane (GO: 0031224) with 28 DEGs, and virion (GO: 0019012) and virion part (GO: 0044423) with eight DEGs, respectively (Fig. 3a).

There were 360 DEGs in the Race15-LN vs. Race15-CK group, 142 of which belonged to 29 GO groups based on biological processes, 161 of which belonged to 16 groups based on molecular functions, and 57 of which belonged to 28 GO groups based on cellular components. We further identified over-represented GO term categories (*p* < 0.05) in the DEGs of the Race15-LN vs. Race15-CK group and classified these terms into 45 categories. For biological processes, the dominant categories were single-organism process (GO: 0044699) with 59 DEGs, single-organism cellular process (GO: 0044763) with 40 DEGs, single-organism localization (GO: 1902578) with 26 DEGs, single-organism transport (GO: 0044765) with 26 DEGs, and transport (GO: 0006810) with 26 DEGs. For molecular functions, the dominant categories were catalytic activity (GO: 0003824) with 45 DEGs, transferase activity (GO: 0016740) with 22 DEGs, oxidoreductase activity (GO: 0016491) with 24 DEGs, transferase activity and transferring phosphorus-containing groups (GO: 0016772) with 16 DEGs, transporter activity (GO: 0005215) with 24 DEGs, and transmembrane transporter activity (GO: 0022857) with 15 DEGs. For cellular components, the dominant categories were membrane (GO: 0016020) with 29 DEGs, integral component of membrane (GO: 0016021) with 17 DEGs, intrinsic component of membrane (GO: 0031224) with 17 DEGs, and virion part (GO: 0044423) and virion (GO:0019012) with four DEGs, respectively (Fig. 3b).

**Fig. 4:**
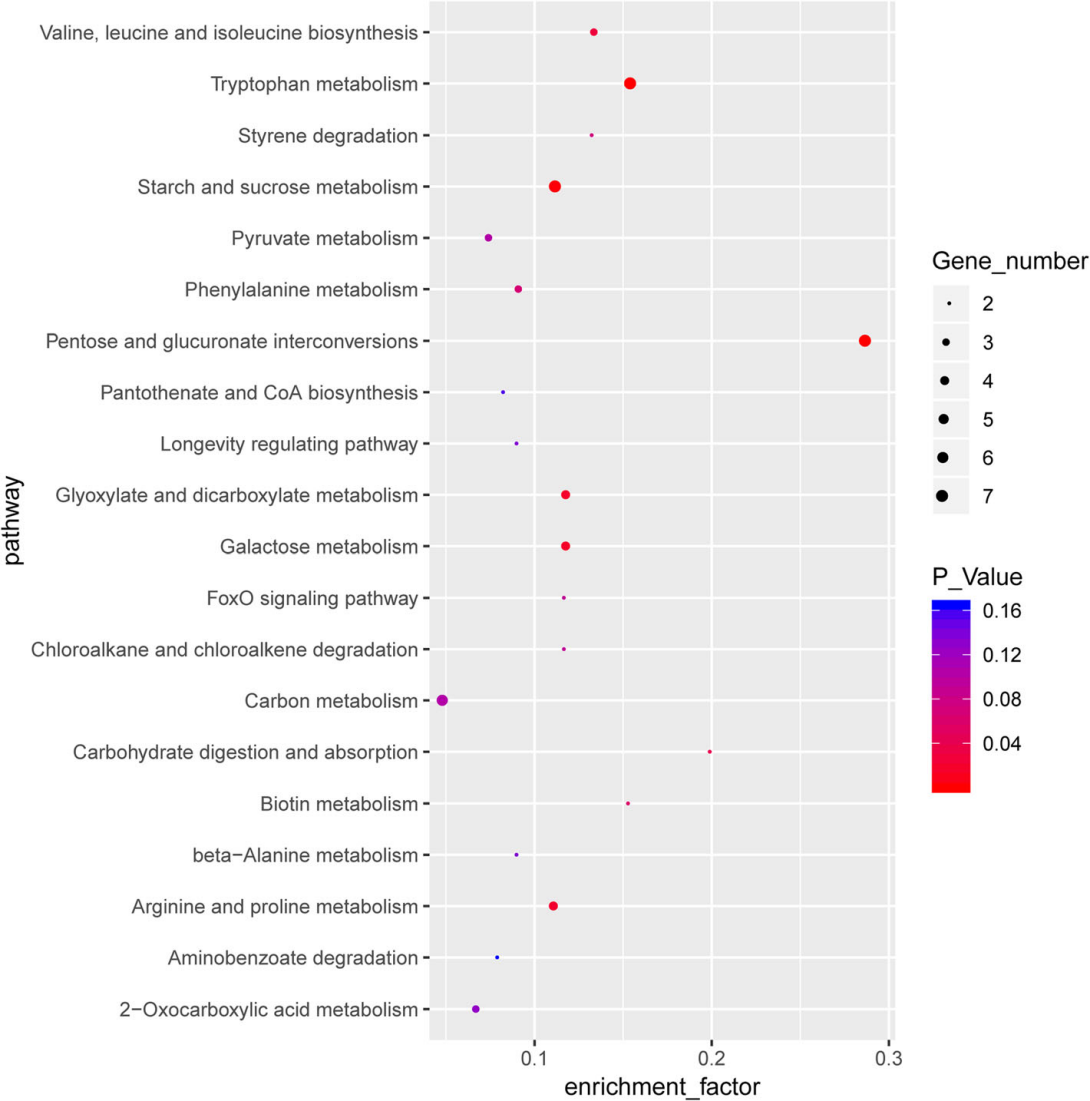
The most significantly enriched KEGG terms of the differentially expressed genes from the Race1-LN vs. Race1-CK group

To further investigate the functions, 136 DEGs were mapped to 70 pathways in the Kyoto Encyclopedia of Genes and Genomes (KEGG) database (Additional file 5: Table S5). In the Race1-LN vs. Race1-CK group, the functions of the transcripts were found to be mainly associated with glyoxylate and dicarboxylate metabolism, tryptophan metabolism, carbon metabolism, FoxO (forkhead box protein O) signaling pathway, valine, leucine, and isoleucine biosynthesis, naphthalene degradation, pentose and glucuronate interconversions, starch and sucrose metabolism, galactose metabolism, arginine and proline metabolism, and tryptophan metabolism (Fig. 4).

**Fig. 5:**
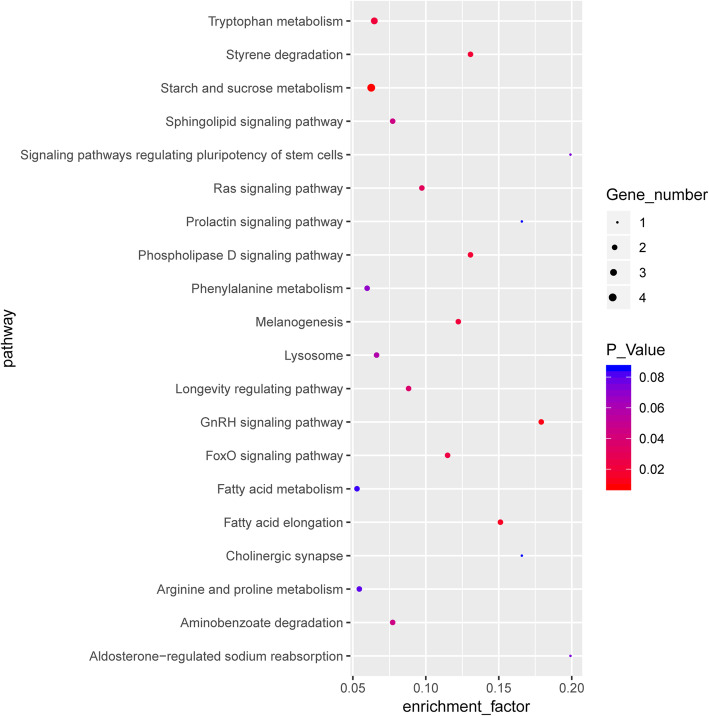
The most significantly enriched KEGG terms of the differentially expressed genes from the Race15-LN vs. Race15-CK group

In the Race15-LN vs. Race15-CK group, 106 DEGs were mapped to 83 pathways in the KEGG database (Additional file 6: Table S6). The functions of the DEGs were found to be mainly associated with tryptophan metabolism, fatty acid elongation, styrene degradation, aminobenzoate degradation, phenylalanine metabolism, arginine and proline metabolism, fatty acid metabolism, starch and sucrose metabolism, signaling pathways regulating pluripotency of stem cells, ErbB signaling pathway, and gap junction (Fig. 5). The significant top-10 pathways of both strains, including ‘starch and sucrose metabolism’, ‘tryptophan metabolism’, and ‘phenylalanine metabolism’, shared the same significant terms.

### Comparative analysis of unique DEGs associated with strain virulence

We combined the DEGs of the four comparison groups, resulting in a total of 923 genes, which were then annotated into virulence-related databases (Additional file 7: Table S7). A total of 46 genes were identified as CAZymes among the 923 DEGs. These were sorted into super families, with 22 glycoside hydrolases (GHs) being the most abundant, followed by nine auxiliary activities (AAs), six carbohydrate-binding modules (CBMs), four glycosyl transferases (GTs), three carbohydrate esterases (CEs), and two polysaccharide lyases (PLs). The annotation results showed that 42 of the 923 DEGs were characterized as known genes proven to affect the outcome of pathogen–host interactions in various pathogenic fungi. In addition, 42 unigenes homologous to PHI (pathogen host interactions) genes associated with reduced virulence (21), unaffected pathogenicity (nine), loss of pathogenicity (six), and increased virulence (six) were considered to be pathogenicity determinants for *C. sojina*. Sixty-two of the 923 DEGs were predicted as involved in secondary metabolic processes, including 34 non-ribosomal peptide synthases (NRPS), 17 type-I polyketide synthases (T-I PKS), four T-I PKS-NRPS, one terpene, and six others. According to our screening results, 129 genes were predicted as secretory proteins.

The virulence-related DEGs of the four comparison groups were analyzed individually. Earlier, we found that the DEGs in Race15-CK vs. Race1-CK were greater than in Race15-LN vs. Race1-LN. The results indicated that there were distinct differences between the two strains before nitrogen starvation stress, and thus we further analyzed these virulence-related DEGs in the Race15-CK vs. Race1-CK group. A total of 54 virulence-related DEGs were identified, including nine in PHI, 31 in Secretory Protein, six in CAZymes, and 15 in Secondary Metabolism. Among the pathogenicity genes, two DEGs significantly, including scytalone dehydratases (SCD) and 1,3,8-trihydroxynaphthalene reductase genes (THR) (PHI: 2312 and PHI: 2313), were noted and were annotated as associated with increased virulence in the PHI database and were specifically upregulated. These genes are all associated with the melanin pathway, and melanin is believed to enhance the survival and competitive abilities of fungi in certain environments [19] and are thus important virulence factors for certain plant pathogenic fungi [20].

Although the two strains with differing virulence differed prior to nitrogen starvation treatment, the DEGs after nitrogen starvation treatment may also partially account for the difference in virulence. We therefore compared the virulence-related DEGs in the Race15-LN vs. Race1-LN group. Among the 31 pathogenicity-related DEGs, one gene *Vtc4* (PHI: 3457) was notable and was annotated as associated with increased virulence in PHI and was specifically upregulated. *Vtc4* is involved in the storage and transport of polyphosphates (polyP) in fungal vacuoles, and phosphate can directly influence the morphology of *Ustilago maydis* in response to lipids, and under increased phosphate levels, filamentation is enhanced [21]. The deletion of *Vtc4* significantly reduced the amount of polyP stored in the vacuole, resulting in decreased virulence and slowed symptom development in maize [22]. A CAZy enzyme, namely, β-1,3-glucanase (EC 3.2.1.39), is also involved in the interaction of fungal pathogens and plants, degrading the β-1,3-glucan of the host cell wall during pathogen invasion [23].

### WGCNA results

**Fig. 6:**
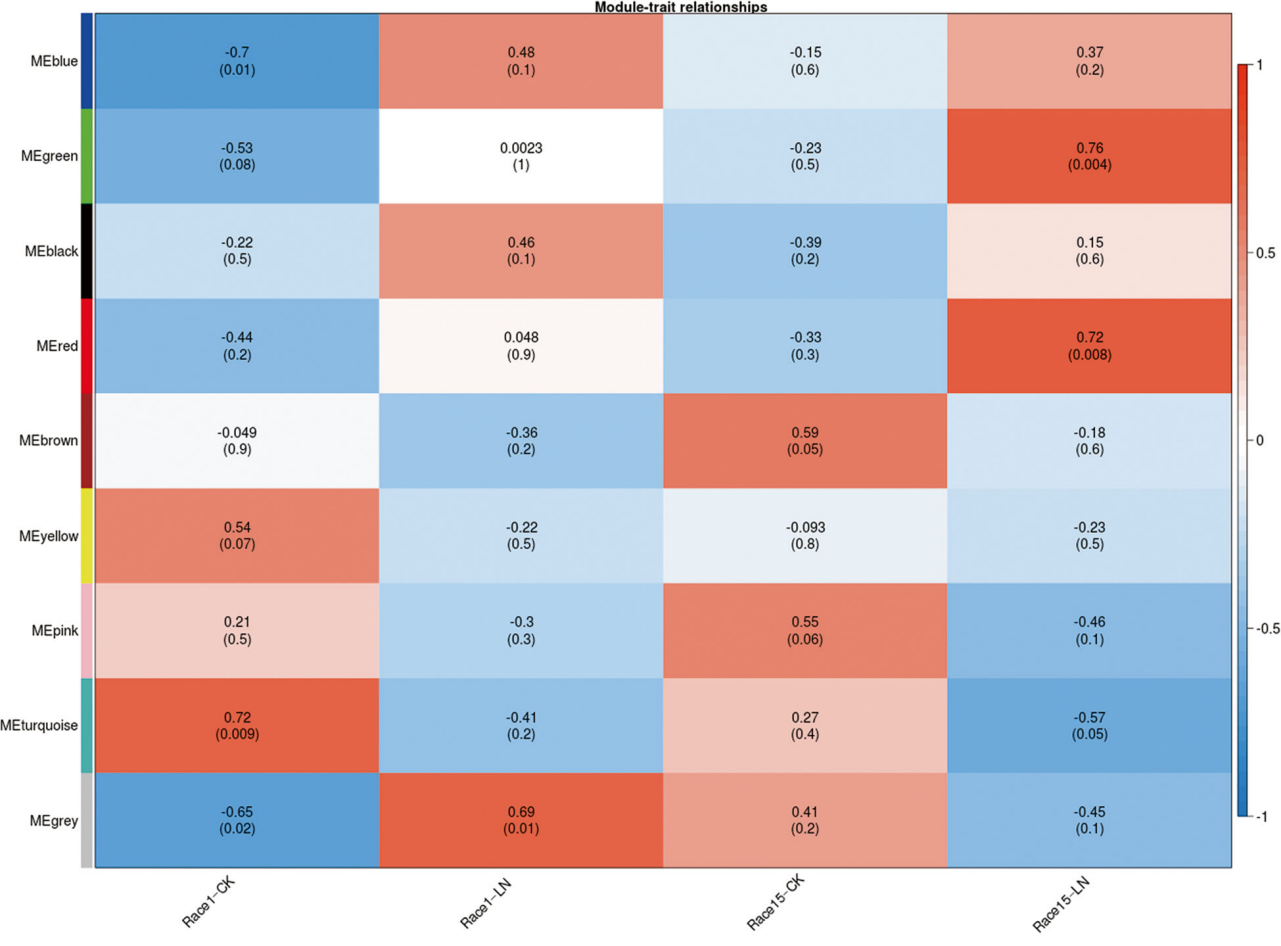
WGCNA of the transcript changes in the highly (Race15) and mildly (Race1) virulent strains. **a** Module trait correlation analysis showed that two modules were correlated with the high virulence of *Cercospora sojina*after infection. Each module has a space with each trait. The upper part of the space is the correlation coefficient between the module and the trait. The lower part of the space is a *p*-value, which represents the significance of the correlation coefficient. Red indicates positive correlation and blue indicates negative correlation. **b** Expression level of the ME gene in the green module. **c** Expression level of the ME gene in the red module. **d** Expression level of the ME gene in the turquoise module

To identify specific virulence-related unique genes, the Fragments Per Kilobase of gene model per Million mapped reads (FPKM) data of the two strains in different media were subjected to WGCNA. When the soft threshold was 28, the square of the correlation coefficient between log (k) and log (p(k)) was close to 0.85 and reached the platform period. Finally, nine co-expression modules were named after randomly assigning colors by dynamic tree cutting. Gray modules represent genes that could not be assigned to any one module. Two of the modules (red and green modules, *p* < 0.01) were relevant to the highly virulent strain under nitrogen starvation stress (Race 15-LN) (Fig. 6). The genes of this module were highly expressed in three Race15-LN samples and could be related to the function and development of Race15 under nitrogen starvation stress. In addition, considering that there were greater DEGs between the CK group than the nitrogen starvation treatment group, this difference may be related to sensitivity to stress. The turquoise module exhibited a strong positive correlation with Race1 in the CK group, and thus, the module genes may be related to sensitivity to nitrogen starvation. Therefore, the three modules (red, green, and turquoise) were annotated for further analysis in PHI, CAZymes, secretory protein, and secondary metabolic processes databases.

In the green module, GO enrichment analysis showed that the genes were enriched in single-organism process, biological regulation, metabolic process, cellular process, catalytic activity, binding, membrane, and organelle. The KEGG enrichment results showed that the genes were enriched in a two-component system, regulation of mitophagy, and the MAPK signaling pathway. In module ‘green’, 18 of the 48 genes were associated with virulence. However, most of the unique genes in this module were annotated in the CAZymes database, including α-mannosyltransferase, β-1,3-glucanase, β-1,4-xylan, and NRPS. Some of these modules also bind β-1,3-glucan, β-1,3-1,4-glucan, and endo-β-1,4-glucan. The gene *Vtc4* was also in the green module and was upregulated in the Race15-LN vs. Race1-LN group (Additional file 8: Table S8).

The red module was the most concentrated module of the virulence-related DEGs associated with Race15. GO enrichment analysis indicated that the genes were enriched in metabolic process, single-organism process, cellular process, catalytic activity, and binding, etc. The KEGG enrichment results showed that the genes were found to be mainly associated with starch and sucrose metabolism, carbohydrate digestion and absorption, quorum sensing, pentose and glucuronate interconversions, and amino sugar and nucleotide sugar metabolism. In total, 28 genes belonged to the module ‘red’, of which 18 were related to the virulence of strain Race15, including cellobiose dehydrogenase, endo-β-1,4-glucanas, endo-β-1,4-xylanas, α-amylase, and pectate lyase (Additional file 9: Table S9). In the red module, cellobiose dehydrogenase was specifically upregulated only in the Race 15-LN vs. Race15-CK group. This inducible enzyme participates in lignocellulose degradation by various phytopathogenic fungi and has a significant role in the early progress of wood degradation, as investigated in several basidiomycete fungi transcriptome studies [24].

In the turquoise module, GO enrichment analysis indicated that the DEGs were enriched in single-organism process, localization, cellular process, metabolic process, transporter activity, catalytic activity, binding, membrane, and membrane part. The KEGG enrichment results showed that the genes were found to be mainly associated with 100 related pathways, such as arginine and proline metabolism, aminobenzoate degradation, tryptophan metabolism, phenylalanine metabolism, and fatty acid elongation. There were 273 genes in the turquoise module, of which 55 were associated with virulence (Additional file 10: Table S10). Furthermore, more DEGs were associated with reduced virulence, loss of pathogenicity, and unaffected pathogenicity.

### Validation by qRT-PCR

**Fig. 7:**
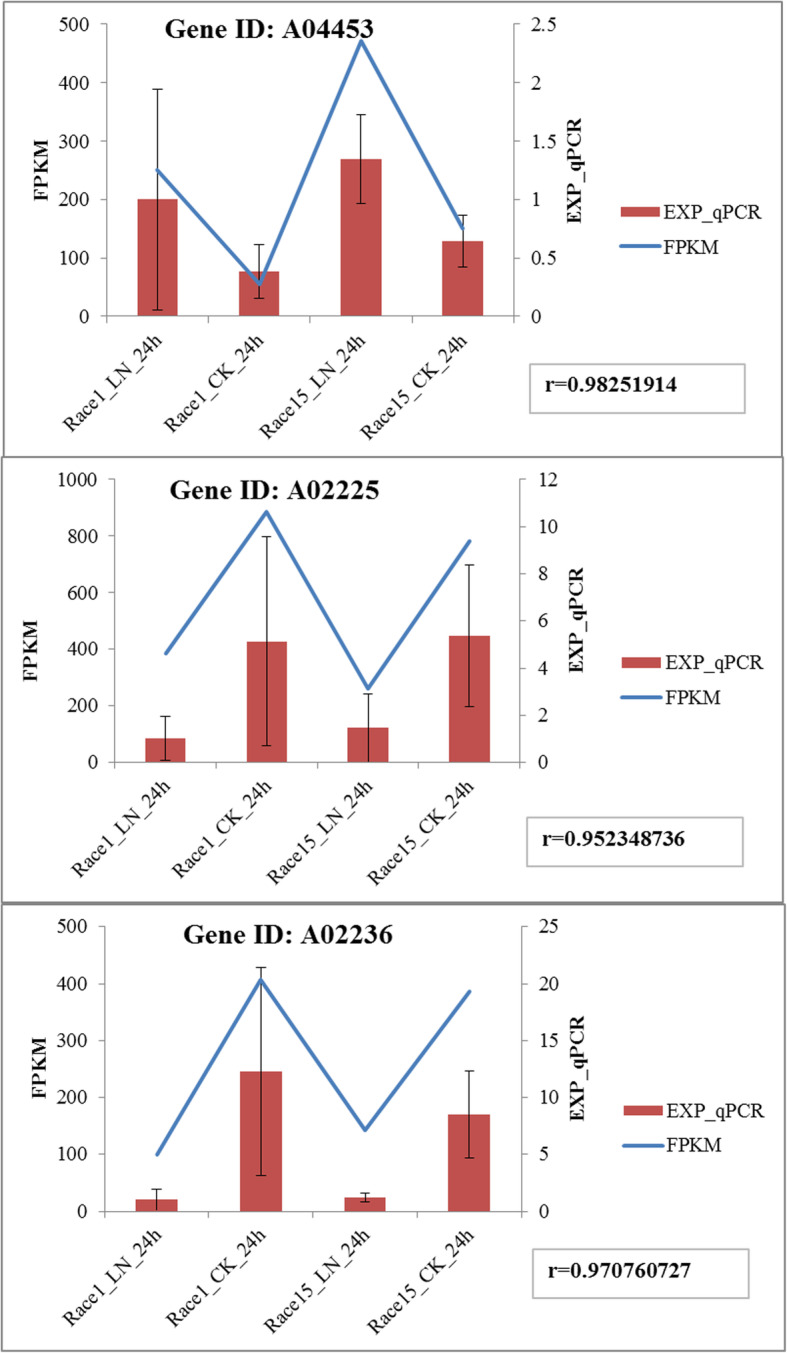
Verification results of the differential gene expression by qRT-PCR

In order to verify the accuracy of the high-throughput sequencing results, three genes were randomly selected for verification of the expression levels, including linoleate glycol synthase (gene id: A04453), cell wall integrity signaling protein Lsp1 / Pil1 (gene id: A02225), and ZIP zinc / iron transport family (gene id: A02236). The RNA samples used for the qRT-PCR verification were processed in the same way as the high-throughput sequencing RNA samples. The average relative expression of each group obtained by qRT-PCR was consistent with the average FPKM value of each group obtained by sequencing. The two groups possessed a strong positive correlation (*r* > 0.95), implying that the high-throughput sequencing data were reliable (Fig. 7).

## Discussion

Previous research indicated that the cercosporin secreted by *C. sojina* was not present in either infected plant tissue or cultured mycelium using the method applied in other *Cercospora* species [11]. An earlier study [11] found that the *C. sojina* Race 1 genome encodes many PKSs that are involved in pigment biosynthesis. During the early stage of infection, the genes involved in pigment biosynthesis are significantly upregulated after starvation and cyclic adenosine monophosphate complete medium treatments, from which eight cercosporin biosynthesis genes were screened. Amino acid sequence alignments showed that they shared the same tandem order with *C. nicotianae*, implying that the pigments may be related to *C. sojina* virulence. In addition to mycotoxins, pigments are other important secondary metabolites that allow phytopathogenic fungi to successfully invade hosts. Melanin is a multifunctional pigment that is widely present in a variety of fungi. It not only participates in the fungal infection process, but also enhances fungal survival and competitive ability during adversity [25, 26]. Melanin deposits are involved in the structural formation of *M. oryzae* appressorium. Furthermore, blocking of melanin synthesis will result in the inability of fungi to generate the high pressure required for breaching the cuticle and the plant cell wall [27]. In some fungi that do not produce attached cells, such as the appressorium, melanin is also important for infection. It can increase the cell wall toughness of hyphopodia, which develop from vegetative hyphae, and the osmotic pressure of wild melanized hyphopodia is significantly higher than that of albino hyphopodia [28].

The results of this study indicated that there was a difference between the mildly and highly virulent strains prior to nitrogen starvation stress. SCD and THR were both annotated as associated with increased virulence in the PHI database, and some PKS and NRPS were both specifically upregulated. All of these participate in the synthesis of melanin. Current research confirms that a lack of SCD in dihydroxynaphthalene (DHN)-derived melanin biosynthetic pathways leads to a loss of pathogenicity [29]. These melanin biosynthetic genes are expressed early during the germination of the conidia, and a previous study found that after 2 h of conidia incubation, the mRNA of PKS, THR, and SCD began to accumulate [30–32]. THR reductase is also involved in the biosynthesis of fungal DHN-derived melanin. Targeted disruption of the THR gene indicated that it is important for melanin biosynthesis in *Bipolaris oryzae* [33]. A *Colletotrichum lagenarium* melanin-deficient mutant 9141 (Thr-) is defective in converting 1,3,8-trihydroxynaphthalene to vermelone in the melanin biosynthetic pathway, resulting in the formation of nonmelanized appressoria, with little infectivity on cucumber leaves [34]. When inoculating the THR knockout mutant strains of *Curvularia lunata* and *Setosphaeria turcica* on susceptible maize leaves, reduced virulence was detected compared with the wild-type strains [35, 36]. However, some studies have obtained contrary results. Knockout of the SCD and THR genes in *S. sclerotiorum* demonstrated no effect on pathogenicity. Similarly, in *A. alternata,* the melanin-deficient strain did not demonstrate reduced osmotic pressure of the appressoria, and pathogenicity was also not affected. However, the mycelial structure and morphology of *S. sclerotiorum* was found to change. Studies suggest that melanin synthesis in *Sclerotinia* is not completely regulated by these two genes, and other melanin biosynthesis pathways might be involved [37]. The genes have been characterized in relation to UV resistance or complementation in restoring the virulence of *M. grisea*, *S. sclerotiorum,* and *C. lagenarium* [38, 39]. Following knockout of these two genes, the sensitivity of the mutant strain to UV radiation was significantly increased. The two genes that affect melanin synthesis are important for the survival of the fungus during adversity. In the field, the fungus will experience a period of unfavorable conditions, such as low temperature and strong sunlight. Without melanin, survival will be difficult for the fungus.

*Vtc4* was annotated as exhibiting increased virulence in PHI and was specifically upregulated in the Race15-LN vs. Race1-LN group, and it was also identified in the virulence-related green module. It is mainly involved in the storage and transportation of polyP in the vacuoles. Inferior to nitrogen, inorganic phosphate is an important nutrient for many fungal structures and metabolism during development. In some eukaryotes, polyP is involved in phosphate transport, osmotic pressure regulation, and skeletal calcification between mycorrhizal fungi and symbiotic plants [40]. PolyP also has various roles in phosphate and energy storage, cation sequestration and storage, the formation and functions of cell surface structures, the regulation of gene expression and enzyme activities, and adaptation to stress [41]. In the *Vtc4* mutant of *Saccharomyces cerevisiae*, the capacity to accumulate polyP in the vacuole was reduced due to the lack of vacuolar fusion [42]. However, it is unclear how the fungus can access the phosphate, and it is often found that the amount available in the intercellular space of the fungus may be limited. Therefore, it is possible that *U. maydis* must rely on stored intracellular phosphate to provide sufficient phosphate for structural and metabolic utilization [20]. *Vtc4* deletion mutant strains showed reduced virulence in maize seedlings, and the pathogen was unable to proliferate extensively in host tissues [20]. The accumulation and metabolism of polyphosphates are also important for the virulence of *Cryptococcus neoformans*. The deletion of *vtc4* perturbed the formation of melanin and attenuated virulence [43].

WGCNA identified two modules for the DEGs in the four groups as virulence-associated modules with many CAZymes. Fourteen genes for CAZy family GH109 proteins were abundant in the Race1 genome, which is more than in most fungi, including *M. oryzae*, *Botrytis cinerea,* and *Neurospora crassa* [11]. These GH109 family genes contribute to lectin-mediated resistance in soybean. Interestingly, we did not find the GH109 family in the virulence-related red and green modules, and it was also not found in the comparison groups of Race15-LN vs. Race1-LN and Race 15-CK vs. Race 1-CK. This indicated that although the GH109 family is related to the infection of *C. sojina*, it is not the cause of the difference in virulence between Race15 and Race1.

We also found that three and one carbohydrate-binding module 1 (CBM 1) proteins were significantly upregulated in Race15-LN vs. Race15-CK and in Race1-LN vs. Race1-CK, respectively, under nitrogen starvation stress, which is significantly less than in other phytopathogenic fungi, such as *Verticillium dahliae*. The three CBM1 genes were all in the red module related to virulence and are also candidate genes for differences in virulence. CBM1 is mainly derived from fungi and is involved in cell wall hydrolysis. CBM1 anchors the catalytic region of the enzyme to insoluble cellulose [44], enabling it to attach to the plant cell wall, which may improve the efficiency of plant cell wall digestion by the enzyme. Many of the glycoside hydrolases identified in fungi belong to CBM1. CBM1 not only increases the cellulase concentration on insoluble cellulose but also increases the catalytic activity of cellulase [45]. A comparative proteomics study of two cellulolytic fungi found that CBM1 not only targets enzymes to insoluble cellulose but also attaches enzymes to lignin [46]. The lack of CBM1 in the genome of *C. sojina* may be one explanation for the slow infection rate.

β-1,3-Glucanase was significantly upregulated in the Race15-LN vs. Race1-LN group and was also identified in the green module. β-1,3-Glucanases are widely distributed in fungi and have different functions, including critical roles in many physiological processes and various morphogenetic events during fungal development and differentiation [47]. They are also involved in the mobilization of β-1,3-glucan as autolytic enzymes when carbon and energy are depleted [48, 49]. During fungal infection in plants, β-1,3-glucanase degrades β-D-1,3-glucan in the host vascular tissue [50].

We also found NRPSs in the Race15-LN vs. Race1-LN group and in the red and green modules associated with high virulence; however, no T-I PKS were found. The number of T-I PKS in the turquoise module associated with weak virulence was much higher than NRPS, while more NRPS and TPKS were found in Race15-LN vs. Race15-CK and Race1-LN vs. Race1-CK groups. This indicated that *C. sojina* secreted T-I PKS during the infection process*,* but the difference in virulence is most likely related to NRPSs. NRPSs also play an important role in fungal infection, because several NRPS products have been proven to be virulence factors [51]. NRPSs are multifunctional proteins that can synthesize the ribosome-independent production of small peptides. To date, the NRPS method of small peptide biosynthesis has mainly been found in filamentous ascomycete fungi and bacteria, but it has not been detected in plants. Additionally, some NRPSs have been shown to be involved in the synthesis of mycotoxins [51, 52], including HC-toxin produced by *Cochliobolus carbonum* and AM-toxin produced by *A. alternata*. Knockout of the NRPS gene will hinder the synthesis of mycotoxins and lead to a loss of virulence [53, 54]. The whole-genome sequencing analysis of the corn pathogen *Cochliobolus heterostrophus* indicated that it contains a large number of NRPS-encoding genes. After knocking them out individually, it was found that only one NPS6 (NRPS-encoding genes) was closely related to the virulence of the pathogen, causing reduced virulence and increased sensitivity to H_2_O_2_ [55]. The NRPS-deficient mutant of *A. alternata* showed significantly decreased virulence and an albino phenotype on potato dextrose agar (PDA) medium, indicating that melanin synthesis was also affected [56]. It was also found that most fungal siderophores are products of NRPS, and siderophores are essential growth factors for microbial growth [57]. Siderophores also act as a signaling factors, regulating the growth, reproduction, and differentiation of fungi [58].

## Conclusion

This study is the first to analyze the DEGs between highly and mildly virulent *C. sojina* strains. A total of 54 and 31 virulence DEGs between Race 15 and Race 1 were annotated before and after nitrogen starvation treatment, respectively. There were 36 virulence-related DEGs that were identified in two highly virulent modules. The significant nitrogen starvation-responsive DEGs were involved in the synthesis of melanin, polyP storage in the vacuole, lignocellulose degradation, and cellulose degradation during fungal development and differentiation. The qRT-PCR analysis of three DEGs showed that expression was very specific at certain time periods during infection, and some DEGs may play specific roles either early or late in the disease process. Functional analysis of these virulence-related DEGs needs to be carried out to discover the molecular mechanisms of race variation.

## Methods

### Virulence tests and strain growth conditions

The strains were maintained on V8 juice agar medium (30 g V8 juice, 20 g agar, 1000 mL H_2_O) at 25 °C for 10 d before virulence determination. The conidia were scraped lightly by flooding the Petri dishes with sterile distilled water to produce conidial suspensions (6 × 10^4^ conidia mL^− 1^). One trifoliate leaf per soybean seedling at growth stage V2-V3 was inoculated with 0.3 mL conidial suspension. The inoculated plants were then kept in a growth chamber under high humidity at 26–28 °C with a 12 h light cycle for 72 h. Disease severity was evaluated by the lesion size and number of spots after 14 d of inoculation [1]. All of the virulence determinations were repeated at least three times and included 30 plants per treatment. The plant materials used in the current study were collected from the Heilongjiang academy of agricultural sciences, which are public and available for noncommercial purpose.

For transcriptome sequencing, the strains were cultured in nitrogenous medium (1 g K_2_HPO_4_, 0.5 g MgSO_4_·7H_2_O, 0.5 g KCl, 0.01 g FeSO_4_, 3 g NaNO_3_ and 30 g sucrose per L), after 7 d they were transferred into a nitrogen-deficient medium (remove NaNO_3_) for 24 h, and three biological samples were collected for RNA extraction [14].

### Total RNA extraction

Total RNA was extracted from the mycelia using TRIzol reagent (Invitrogen, USA) following the manufacturer’s instructions. The quantity and purity of the RNA were assessed by 1% agarose gel electrophoresis and Nanodrop2000 (Thermo Fisher ScientificWaltham, MA, USA). RNA quantification was conducted using a Qubit® 3.0 Fluorometer (Thermo Fisher Scientific), and RNA integrity was assessed using the RNA Nano 6000 Assay Kit of the Bioanalyzer 2100 system (Agilent Technologies, CA, USA).

### Transcriptome sequencing, quality control, and mapping

A total of 3 μg RNA per sample was used as input material for the RNA sample preparations. Subsequently, sequencing libraries were generated using the NEBNext® Ultra™ RNA Library Prep Kit for Illumina® (NEB, USA) following the manufacturer’s recommendations [59]. Briefly, mRNA was purified from total RNA using poly-T oligo-attached magnetic beads for eukaryotes. This was mixed with the fragmentation buffer, and the mRNA was fragmented into short fragments. The library was sequenced using the Illumina HiSeq.2000 platform [59]. Fastx-toolkit software was used to filter the raw reads by removing reads with adapters, those with more than 5% unknown bases (N), and those with low quality reads (quality less than 15% and greater than 20% in a read). HISAT2 v2.0.1 [60] was used to align the clean reads to the reference genome of *C. sojina* (Race 15), and the sequence aligned to the unique position of the reference genome was extracted for subsequent analysis. StringTie v1.3.1 (https://ccb.jhu.edu/software/stringtie/) was used to splice and assemble sequences to obtain more complete and accurate genes and transcripts. The default parameters were applied in the above software.

### Identification of DEGs and bioinformatics analysis

HTSeq v0.6.1 (http://www-huber.embl.de/users/anders/HTSeq/doc/index.html) was used to quantify the gene expression levels in response to nitrogen starvation stress. The FPKM of each gene was then calculated based on the gene length and reads count mapped to this gene [61]. Differential expression analysis was performed using the edgeR package in R (v3.3.2) [62], and three biological replicates of RNA-Seq experiments were analyzed separately. A gene that met the difference in expression fold |log_2_(fold change)| > 1 and FDR < 0.01 (false discovery rate) was defined as differentially expressed. The resulting *p*-values were adjusted using the Benjamini and Hochberg’s approach for controlling the FDR [63]. Genes with an adjusted *p* adj (*p*-adjusted) < 0.05 found by edgeR were assigned as differentially expressed. GO enrichment analysis of DEGs was implemented by the GO seq R package [64], and GO terms with corrected *p*-values <0.05 were considered to be significantly enriched. KOBAS (KOBAS, London, UK) was used to test the statistical enrichment of the DEGs in KEGG pathways.

### Comparative analysis of unique DEGs associated with virulence

Secondary metabolites and secreted proteins are involved in infestation, colonization, and lesion formation at different fungal infection stages. PHI-base catalogs experimentally verify the pathogenicity, virulence, and effector genes from fungal and hosts as well as the CAZymes involved in plant cell wall degradation during infection. Therefore, we combined the DEGs of the four comparison groups and annotated them in these four databases related to virulence. SignalP v4.1 was used to predict the secretory signal peptides of proteins [65], and transmembrane helix prediction was determined using TMHMM v2.0c [66]. AntiSMASH v2.0.2 (fungi) [67] was used to predict the putative secondary metabolites and biosynthetic gene clusters.

Putative pathogenic genes were identified by searching in the PHI database with Blastp (E ≤ 1 × 10^− 5^) against protein sequences [68]. CAZymes were identified and classified into different CAZyme families using the CAZymes Analysis Toolkit (E ≤ 1 × 10^− 5^) [69] and were annotated using dbCAN [70].

### WGCNA

The FPKM values of the DEGs from the four comparison groups were used as the starting data, and the WGCNA R software package (R 1.66) [71] was used for analysis based on Pearson’s correlation coefficient for WGCNA. The correlation coefficients between genes were weighted by a power function to obtain a scale-free network. The soft threshold (power) chosen for this experiment was 28, which obtained a square of the correlation coefficient of around 0.85. Gene dendrograms were obtained by average linkage hierarchical clustering, with the color row underneath the dendrogram indicating the module assignment determined by dynamic tree cutting.

### Validation of RNA-Seq by qRT-PCR

To validate the results of the RNA-Seq data, a total of three unigenes were randomly selected for qRT-PCR analysis. The qRT-PCR primer pairs were designed using Premier 3.0 (Premier Biosoft, Palo Alto, CA, USA), and the internal reference gene was A01457 (GAPDH). The first strand of cDNA was synthesized by TUREscript 1st Stand cDNA Synthesis Kit (Aidlab, China), using 1 μg RNA as the template. The qRT-PCR assay was performed using the 2 × SYBR® Green Premix (DBI, Germany) in the qTOWER2.2 (Analytikjena, Germany) real-time quantitative PCR system. Each experiment was performed in three replicates, and the difference multiple was calculated using the 2 ^–∆∆Ct^ method.

## Supplementary information

**Additional file 1: Table S1.** Differentially expressed genes in the Race1-LN vs. Race1-CK group.

**Additional file 2: Table S2.** Differentially expressed genes in the Race15-LN vs. Race15-CK group.

**Additional file 3: Table S3.** Differentially expressed genes in the Race15-LN vs. Race1-LN group.

**Additional file 4: Table S4.** Differentially expressed genes in the Race15-CK vs. Race1-CK group.

**Additional file 5: Table S5.** KEGG pathway analysis of the differentially expressed genes in the Race1-LN vs. Race1-CK group.

**Additional file 6: Table S6.** KEGG pathway analysis of the differentially expressed genes in the Race15-LN vs. Race15-CK group.

**Additional file 7: Table S7.** Functional annotation of differentially expressed genes.

**Additional file 8: Table S8.** Putative virulence-related genes in the green module.

**Additional file 9: Table S9.** Putative virulence-related genes in the red module.

**Additional file 10: Table S10.** Putative virulence-related genes in the turquoise module.

## Data Availability

The RNA-Seq data 12 samples of two strains are available in the Sequence Read Archive (SRA) repository of National Center for Biotechnology Information (NCBI) with the accession numbers PRJNA610974.

## Abbreviations

DEGs: Differentially expressed genes
CBM: Carbohydrate-binding module
CE: Carbohydrate esterase
GH: Glycoside hydrolase
WGCNA: Weighted gene co-expression network
NRP: Nonribosomal peptides
NRPS: Non-ribosomal peptide synthase
SCD: Scytalone dehydratase
THR: 1,3,8-trihydroxynaphthalene reductase genes
polyP: Polyphosphates
CAZymes: Carbohydrate-Active enzymes
